# Antimicrobial resistance in *Neisseria gonorrhoeae* isolates and gonorrhoea treatment in the Republic of Belarus, Eastern Europe, 2009–2019

**DOI:** 10.1186/s12879-021-06184-7

**Published:** 2021-06-02

**Authors:** Aliaksandra Aniskevich, Iryna Shimanskaya, Iryna Boiko, Tatyana Golubovskaya, Daniel Golparian, Iryna Stanislavova, Susanne Jacobsson, Aliaksandr Adaskevich, Magnus Unemo

**Affiliations:** 1 Belarusian Medical Academy of Postgraduate Education, Minsk, Belarus; 2 Department of Functional and Laboratory Diagnostics, I. Horbachevsky Ternopil National Medical University, Ternopil, Ukraine; 3 World Health Organization Collaborating Centre for Gonorrhoea and Other Sexually Transmitted Infections (STIs), National Reference Laboratory for STIs, Department of Laboratory Medicine, Clinical Microbiology, Faculty of Medicine and Health, Örebro University, Örebro, Sweden; 4 Mogilev Regional Skin and Venereal Diseases Dispensary, Mogilev, Belarus

**Keywords:** *Neisseria gonorrhoeae*, Antimicrobial resistance, Surveillance, Treatment, Ceftriaxone, Azithromycin, Belarus, Eastern Europe

## Abstract

**Background:**

Limited antimicrobial resistance (AMR) data for *Neisseria gonorrhoeae* are available in Eastern Europe. We investigated AMR in *N. gonorrhoeae* isolates in the Republic of Belarus from 2009 to 2019, antimicrobial treatment recommended nationally, and treatment given to patients with gonorrhoea.

**Methods:**

*N. gonorrhoeae* isolates (*n* = 522) cultured in three regions of Belarus in 2009–2019 were examined. Determination of minimum inhibitory concentrations (MICs) of eight antimicrobials was performed using Etest. Resistance breakpoints from the European Committee on Antimicrobial Susceptibility Testing were applied where available. A Nitrocefin test identified β-lactamase production. Gonorrhoea treatment for 1652 patients was also analysed. Statistical significance was determined by the Z-test, Fisher’s exact test, or Mann-Whitney U test with *p*-values of < 0.05 indicating significance.

**Results:**

In total, 27.8% of the *N. gonorrhoeae* isolates were resistant to tetracycline, 24.7% to ciprofloxacin, 7.0% to benzylpenicillin, 2.7% to cefixime, and 0.8% to azithromycin. No isolates were resistant to ceftriaxone, spectinomycin, or gentamicin. However, 14 (2.7%) isolates had a ceftriaxone MIC of 0.125 mg/L, exactly at the resistance breakpoint (MIC > 0.125 mg/L). Only one (0.2%) isolate, from 2013, produced β-lactamase. From 2009 to 2019, the levels of resistance to ciprofloxacin and tetracycline were relatively high and stable. Resistance to cefixime was not identified before 2013 but peaked at 22.2% in 2017. Only sporadic isolates with resistance to azithromycin were found in 2009 (*n* = 1), 2012 (n = 1), and 2018–2019 (*n* = 2). Overall, 862 (52.2%) patients received first-line treatment according to national guidelines (ceftriaxone 1 g). However, 154 (9.3%) patients received a nationally recommended alternative treatment (cefixime 400 mg or ofloxacin 400 mg), and 636 (38.5%) were given non-recommended treatment.

**Conclusions:**

The gonococcal resistance to ciprofloxacin and tetracycline was high, however, the resistance to azithromycin was low and no resistance to ceftriaxone was identified. Ceftriaxone 1 g can continuously be recommended as empiric first-line gonorrhoea therapy in Belarus. Fluoroquinolones should not be prescribed for treatment if susceptibility has not been confirmed by testing. Timely updating and high compliance with national evidence-based gonorrhoea treatment guidelines based on quality-assured AMR data are imperative. The need for continued, improved and enhanced surveillance of gonococcal AMR in Belarus is evident.

## Background

Gonorrhoea, etiological agent: *Neisseria gonorrhoeae*, is one of the most common sexually transmitted infections (STIs) worldwide [1–3]. According to the World Health Organization (WHO), in 2016 86.9 million new adult cases of gonorrhoea were estimated [2]. In the Republic of Belarus the incidence of reported gonorrhoea (cases per 100,000 inhabitants) has substantially decreased, i.e., from 63 in 2005 to 44.4 in 2009, and finally, to 10.5 in 2018 [4]. The incidence of gonorrhoea in Belarus and many other East European countries is most likely underestimated because of limited testing (especially pharyngeal and rectal samples), insufficient use of nucleic acid amplification tests, incomplete reporting using multiple methods and epidemiological surveillance [5–7].

Alarmingly, the level of antimicrobial resistance (AMR) in *Neisseria gonorrhoeae* to a wide range of drugs has increased dramatically worldwide over the past decades, including first-line treatment [3, 5, 8–11]. Ceftriaxone, an extended-spectrum cephalosporin (ESC), is the last option for first-line empiric monotherapy internationally. It is a concern that in vitro and clinically decreased susceptibility or resistance to ceftriaxone has been reported from many countries [3, 5, 8–28]. This has resulted in that the WHO global gonorrhoea treatment guidelines and international and national guidelines in Europe, Australia, and Canada recommend antimicrobial combination therapies, mostly ceftriaxone 250–1000 mg × 1 intramuscularly (IM) combined with azithromycin 1–2 g × 1 orally [8, 11, 29, 30]. However, some countries, such as Japan, Ukraine and, since 2019 and 2020, the United Kingdom and USA, respectively, recommend high-dose (1 g except in the USA where 500 mg is recommended) ceftriaxone monotherapy, i.e., when chlamydial infection has been excluded [31–34]. Nevertheless, the first treatment failure with a recommended dual therapy for gonorrhoea was reported in 2016 [27]. In 2018, the first gonococcal strain with ceftriaxone resistance combined with high-level resistance to azithromycin was identified in England [25] and Australia [35].

WHO has developed a global action plan to control the spread and minimise the impact of AMR in *N. gonorrhoeae* [10]. One of its key strategies is to globally enhance quality-assured gonococcal AMR surveillance; to identify emerging AMR, monitor AMR trends, and ensure effective patient management by timely and evidence-based refinements of treatment guidelines. In the European Union/European Economic Area (EU/EEA) the European Gonococcal Antimicrobial Surveillance Programme (Euro-GASP) has been monitoring the patterns of AMR since 2004 [12, 36–38]. It is a grave concern that gonococcal AMR surveillance is dreadfully limited [5, 6] in the non-EU/EEA countries of the WHO European Region, and only available in Ukraine, Belarus, and Russia [33, 39–44].

The empirical treatment of gonococcal infections in Belarus is guided by the Clinical protocol for the diagnosis and treatment of patients with STIs and approved by the order of the Ministry of Health of the Republic of Belarus 10/29/2009, No. 1020 (below referred to as ‘2009 Belarusian national gonorrhoea guideline’) [45]. For treatment of uncomplicated gonorrhoea, recommended first-line therapy is ceftriaxone 1 g × 1 IM. Recommended alternative regimens are cefixime 400 mg × 1 orally or spectinomycin 2 g × 1 IM for men and 4 g × 1 IM for women or ofloxacin 400 mg × 1 orally, or lomefloxacin 800 mg × 1 orally [45]. To treat complicated gonorrhoea ceftriaxone 1 g IM or IV every 24 h for 7 days is the recommended first-line therapy. Recommended alternative regimens are spectinomycin 2 g IM every 12 h for 7 days or ofloxacin 200 mg orally every 12 h for 7–14 days, or lomefloxacin 400 mg orally every 24 h for 7–14 days [45]. Notable, spectinomycin is currently not available throughout Belarus [46].

The aims of this study were to 1) analyse the trends in *N. gonorrhoeae* AMR levels in Belarus (2009–2019), 2) review the antimicrobial treatments recommended nationally as well as compliance with these recommendations in Belarus, and 3) provide quality-assured gonococcal AMR data for informing the 2009 Belarusian national gonorrhoea guideline [45].

## Methods

### Study population

Gonorrhoea patients (*n* = 522) diagnosed in accordance with standard care at the following STI Healthcare Institutions were included in the study: Mogilev Regional Dermatovenerologic Dispensary, Mogilev (*n* = 409), Minsk City Clinical Dermatovenerologic Dispensary, Minsk (*n* = 83), and Vitebsk Regional Dermatovenerologic Dispensary, Vitebsk (*n* = 30) in Belarus, September 2009–June 2015 and July 2017–December 2019. For a description of longitudinal AMR data, this cohort includes previously published data (273 patients) from 2009 (*n* = 80) [39] and 2010–2013 (*n* = 193) [40]. Cervical and urethral specimens from females and males, respectively, were collected in accordance with standard care and delivered to the bacteriological laboratory in Amies Transport Medium with Charcoal (Research and production center Chemmedsynthsis, Minsk, Belarus). Sex and age of the patients were recorded but no patient identification data. Exclusion criterium was: not confirmed gonococcal infection. All patients and their sexual partners were to be treated in accordance with the 2009 Belarusian national gonorrhoea guideline [45].

### Culture of *Neisseria gonorrhoeae*

All urogenital swabs were inoculated on the Chocolate agar™ + PolyViteX VCAT3 plates (bioMerieux, Marcy-l’Etoile, France), which were subsequently incubated in 5 ± 1% CO_2_-enriched humid atmosphere at 36 ± 1 °C for 24 h and, if negative, for an additional 24 h. Suspected gonococcal colonies were verified as *N. gonorrhoeae* based on identification of microscopy (Gram-negative diplococci), oxidase reaction, a Vitek® 2 automatic bacteriological analyser (bioMerieux, Durham, NC, USA) with Vitek® 2 NH ID cards (bioMerieux, Marcy-l’Etoile, France), a polymerase chain reaction assay (AmpliSens *Neisseria gonorrhoeae*-screen-Fl; InterLabServices, Moscow, Russia), and matrix-assisted laser desorption-ionisation time-of-flight mass spectrometry (MALDI-TOF MS; Microflex LT, Bruker Daltonik, Bremen, Germany), according to the manufacturer’s instructions.

The isolates were stored in accordance with standard care in a liquid preservation medium containing trypticase-soy broth, yeast extract, agar, and horse serum in liquid nitrogen (− 196°С) or a low-temperature freezer (− 80 °С).

### Antimicrobial susceptibility testing

At the WHO Collaborating Centre for Gonorrhoea and other STIs, Sweden, the minimum inhibitory concentrations (MICs; mg/L) of ceftriaxone, cefixime, azithromycin, spectinomycin, ciprofloxacin, tetracycline, benzylpenicillin, and gentamicin were determined by Etest (bioMerieux, Marcy-l’Etoile, France), following the manufacturer’s instructions and as described previously [33, 39, 40]. Whole MIC dilutions were used for interpretation and clinical susceptibility (S) and resistance (R) breakpoints stated by the European Committee on Antimicrobial Susceptibility Testing (EUCAST) [47] were applied, where available. For azithromycin, no clinical breakpoints exist, and the EUCAST azithromycin epidemiological cut-off value (ECOFF) of MIC > 1 mg/L [47] was applied to indicate isolates with azithromycin resistance determinants (considered as azithromycin resistant below). For gentamicin, breakpoints from a previous publication were used [48]. The Nitrocefin test (Oxoid, Basingstoke, England) was used to identify β-lactamase producing gonococcal strains. For quality controls of the antimicrobial susceptibility testing, the 2016 WHO *N. gonorrhoeae* reference strains [49] were used.

### Treatment of gonorrhoea

The treatment of patients with gonorrhoea (*n* = 1652) diagnosed in accordance with standard care at the Minsk City Clinical Dermatovenerologic Dispensary in 2013–2018 (*n* = 749) and Mogilev Regional Dermatovenerologic Dispensary in 2010–2019 (*n* = 903) were analysed. Uncomplicated and complicated gonorrhoea cases were defined according to the international statistical classification of diseases and related health problems [50]. Gonorrhoea treatment compliance with the 2009 Belarusian national gonorrhoea guideline [45] was evaluated.

### Statistical analysis

Statistical analysis was performed using MedCalc Statistical Software v18.11.3 (MedCalc Software bvba, Ostend, Belgium). The 95% confidence interval (95% CI) was calculated using the exact binomial distribution. The Z-test, Fisher’s exact test, and Mann-Whitney U test were used to compare groups, as appropriate. The level of significance was set at P<0.05.

## Results

### Patients and *Neisseria gonorrhoeae* isolates

*N. gonorrhoeae* isolates (one per patient) from urogenital sites of 522 patients were examined: 430 (82.4%) males, 84 (16.1%) females and eight (1.5%) with sex not reported. The isolates were cultured in Mogilev (78.4%, 409/522), the capital city Minsk (15.9%, 83/522), and Vitebsk (5.7%, 30/522) in 2009 (*n* = 81), 2010 (*n* = 72), 2011 (*n* = 6), 2012 (*n* = 75), 2013 (*n* = 101), 2014 (*n* = 56), 2015 (*n* = 17), 2017 (*n* = 36), in 2018 (*n* = 19), and 2019 (*n* = 59).

Demographic data (sex and age) were available from 514/522 (98.5%) patients. Briefly, the median age for men was 25 years (range 16–61 years) and for women, also 25 years (range 16–74 years). The sex and age distributions were similar in 2009–2019. The median male-female ratio was 5.9 (range 1.8–7.6), with decreasing trends over time.

### Antimicrobial susceptibility of *Neisseria gonorrhoeae* isolates (*n* = 522) from Belarus, 2009–2019

The overall antimicrobial susceptibility of all *N. gonorrhoeae* isolates (*n* = 522) is summarised in Table 1.

**Table 1:** Antimicrobial susceptibility in *Neisseria gonorrhoeae* isolates (*n* = 522) from Minsk, Mogilev, and Vitebsk, Belarus, 2009–2019

Antimicrobials	Susceptible (S)/Susceptible, increased exposure (I)/Resistant (R), %^**a**^							
	2009^**b**^	2010-2011^**b**^	2012^**b**^	2013^**b**^	2014–2015	2017	2018–2019	Total^**b**^
	*n* = 81	*n* = 78	*n* = 75	*n* = 101	*n* = 73	*n* = 36	*n* = 78	*n* = 522
CRO	100/NA/0	100/NA/0	100/NA/0	100/NA/0	100/NA/0	100/NA/0	100/NA/0	100/NA/0
CFM	100/NA/0	100/NA/0	100/NA/0	98.0/NA/2.0	100/NA/0	77.8/NA/22.2	94.9/NA/5.1	97.3/NA/2.7
AZM	98.8/NA/1.2	100/NA/0	98.7/NA/1.3	100/NA/0	100/NA/0	100/NA/0	97.4/NA/2.6	99.2/NA/0.8
SPC	100/NA/0	100/NA/0	100/NA/0	100/NA/0	100/NA/0	100/NA/0	100/NA/0	100/NA/0
CIP	65.4/0/34.6	64.1/2.6/33.3	78.7/0/21.3	72.3/0/27.7	90.4/0/9.6	69.4/0/30.6	83.3/0/16.7	74.9/0.4/24.7
PEN	29.6/60.5/9.9	−/−/−	62.6/30.7/6.7	59.4/31.7/8.9	80.8/16.4/2.8	55.6/33.3/11.1	51.3/44.9/3.8	56.3/36.7/7.0
TET	55.5/21/23.5	56.4/10.3/33.3	56/9.3/34.7	62.4/8.9/28.7	76.7/5.5/17.8	69.4/0/30.6	61.5/11.5/26.9	61.9/10.3/27.8
GEN	72.8/27.2/0	97.4/2.6/0	58.7/41.3/0	40.6/59.4/0	67.1/32.9/0	55.6/44.4/0	87.2/12.8/0	68.4/31.6/0

*CRO* Ceftriaxone, *CFM* Cefixime, *AZM* Azithromycin, *SPC* Spectinomycin, *CIP* Ciprofloxacin, *PEN* Benzylpenicillin, *TET* Tetracycline, *GEN* Gentamicin, *NA* Not applicable, −, not tested

^a^The clinical breakpoints (susceptible, resistant) were as follows: ceftriaxone and cefixime (MIC≤0.125 mg/L, MIC>0.125 mg/L), ciprofloxacin (MIC≤0.032 mg/L, MIC>0.064 mg/L), azithromycin (MIC≤1 mg/L, MIC>1 mg/L), spectinomycin (MIC≤64 mg/L, MIC>64 mg/L), benzylpenicillin (MIC≤0.064 mg/L, MIC>1.0 mg/L), tetracycline (MIC≤0.5 mg/L, MIC>1.0 mg/L), and gentamicin (MIC≤4 mg/L, MIC>16 mg/L) [47, 48].

^b^Of the 335 isolates from 2009-2013, 273 have been previously published [39, 40]

Briefly, in 2018–2019 the resistance to tetracycline, ciprofloxacin, cefixime, benzylpenicillin, and azithromycin was 26.9, 16.7, 5.1, 3.8, and 2.6%, respectively. During 2009–2019, the resistance levels were as follows: tetracycline 27.8% (range: 17.8–34.7%), ciprofloxacin 24.7% (9.6–34.6%), benzylpenicillin 7.0% (2.8–11.1%), cefixime 2.7% (0–22.2%), and azithromycin 0.8% (0–2.6%) (Table 1). Only one (0.2%) β-lactamase-producing isolate was found (in 2013). No isolates resistant to ceftriaxone, spectinomycin, or gentamicin were detected (Table 1). However, 14 (2.7%) isolates had a ceftriaxone MIC of 0.125 mg/L, precisely on the resistance breakpoint of MIC > 0.125 mg/L. Additionally, 31.6% of the isolates had a decreased susceptibility to gentamicin.

During 2009–2019, the level of resistance to tetracycline was relatively high and stable. The level of resistance to ciprofloxacin significantly decreased to 9.6% in 2014–2015, but significantly increased to 30.6% in 2017 (*P* < 0.05). The level of resistance to benzylpenicillin non-significantly fluctuated, i.e., from 2.8 to 11.1% over the years. Of note, no resistance to cefixime was found before 2013 (when 2.0% resistance was identified); however, the cefixime resistance then significantly increased to a peak of 22.2% in 2017 (*P* < 0.05). Only sporadic isolates with resistance to azithromycin were found in 2009 (*n* = 1, 1.2%), 2012 (*n* = 1, 1.3%), and 2018–2019 (*n* = 2, 2.6%) (Table 1).

**Fig. 1:**
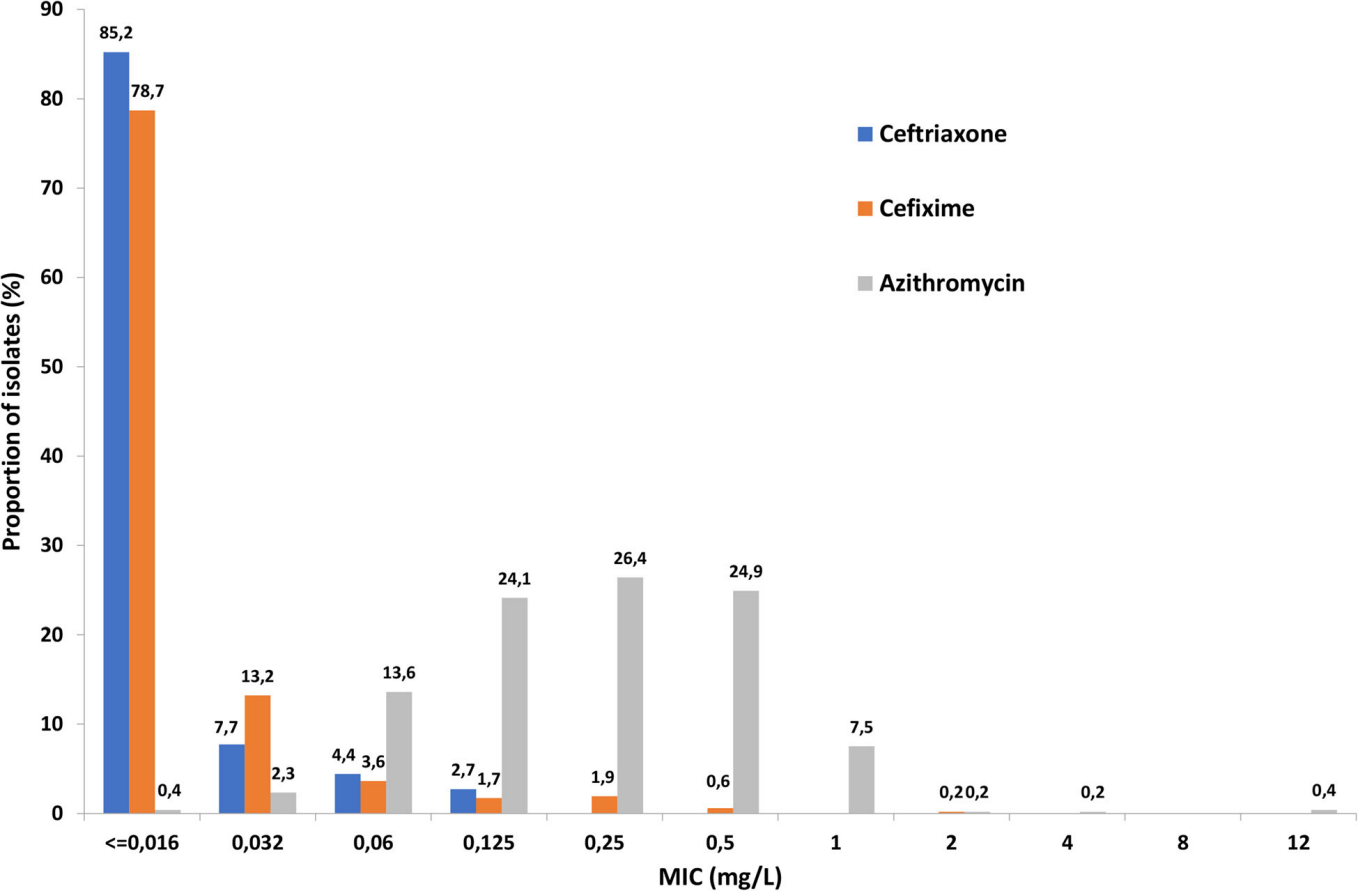
MIC distributions for ceftriaxone, cefixime, and azithromycin for *Neisseria gonorrhoeae* isolates (*n* = 522) from Belarus, 2009–2019

The MIC distributions for ceftriaxone and azithromycin, included in the internationally recommended first-line dual antimicrobial therapy [8, 11, 29], and cefixime are presented in Fig. 1.

In total, 2.7% (14/522) of the isolates had a ceftriaxone MIC of 0.125 mg/L, which is on the ceftriaxone resistance breakpoint [47]. The proportion of isolates with a ceftriaxone MIC of ≤0.016 mg/L was 85.2%, and in general, the annual MIC distribution for ceftriaxone appeared to shift to lower MICs during 2009–2019 (data not shown). For cefixime, 78.7% (411/522) of the isolates had a MIC of ≤0.016 mg/L; however, 2.7% (14/522) of the isolates were resistant to cefixime, highest MIC = 2 mg/L (one isolate in 2013), and additionally, 1.7% (9/522) of the isolates had a cefixime MIC of 0.125 mg/L, i.e., exactly on the cefixime resistance breakpoint [47]. Except for four (0.8%) azithromycin-resistant isolates (MICs 2–12 mg/L), the azithromycin MIC distribution appeared to represent a wild-type distribution.

### Antimicrobial treatment of gonorrhoea patients in Belarus, 2010–2019

Compliance with the treatment of gonococcal infections to the 2009 Belarusian national gonorrhoea guideline [45] at the Minsk City Clinical Dermatovenerologic Dispensary (2013–2018) and the Mogilev Regional Clinical Dermatovenerologic Dispensary (2010–2019) for 1652 patients is described in Table 2.

**Table 2:** Compliance with the 2009 Belarusian national gonorrhoea guideline [45] in Minsk (749 patients, 2013–2018) and Mogilev (903 patients, 2010–2019), Belarus

Prescribed antimicrobials	Minsk No. (%, 95 CI)	Mogilev No. (%, 95 CI)
**Recommended first-line treatment** **[**45**]**		
CRO 1 g × 1 IM (uncomplicated gonorrhoea) or CRO 1 g × 1 IM or IV every 24 h, 7 days (complicated gonorrhoea)^a^	354 (47.3, 43.7–51.0)	508 (56.3, 53.0–59.6)
**Alternative treatment** **[**45**]**		
CFM 400 mg × 1 orally	5 (0.7, 0.2–1.6)	0
OFX 400 mg × 1 orally	26 (3.5, 2.3–5.1)	123 (13.6, 11.4–16.0)
**Non-compliant treatment**		
Non-compliant antimicrobials or doses given, generally higher than recommended	364 (48.6, 45.0–52.3)^b^	272 (30.1, 27.1–33.2)^c^

*No.* Number, *CI* Confidence interval, *CRO* Ceftriaxone, *IM* Intramuscularly, *IV* Intravenously, *CFM* Cefixime, *OFX* Ofloxacin

^a^Frequently, additional antimicrobials were given to treat other non-viral STIs, which had been confirmed or not excluded by appropriate laboratory diagnostics. These included doxycycline, other tetracyclines, different macrolides, fluoroquinolones, oral cephalosporins, penicillins, and nitroimidazoles

^b^More than 1 g of ceftriaxone was quite often given; approximately 10% of the patients received benzylpenicillin, about 5% a tetracycline/macrolide regimen, and one (0.1%) patient was given rifampicin

^c^More than 1 g of ceftriaxone was somewhat frequently given. A benzylpenicillin regimen was rarely given, and for a few patients, > 400 mg ofloxacin or a tetracycline/macrolide regimen was administered

Many antimicrobial treatment regimens were administered (Table 2). Overall, only 862 (52.2%) patients received the recommended first-line treatment (ceftriaxone 1 g (uncomplicated gonorrhoea) or > 1 g (complicated gonorrhoea) as monotherapy or in combination with other antimicrobials) in accordance with the 2009 Belarusian national gonorrhoea guideline [45]. Some 154 (9.3%) patients were given a recommended alternative treatment (cefixime 400 mg or ofloxacin 400 mg as a single oral dose) [45]. Consequently, 38.5% of the patients received treatment not compliant with the 2009 Belarusian national gonorrhoea guideline [45]. Worryingly, notwithstanding the high level of ciprofloxacin resistance, ofloxacin 400 mg × 1 was given to 149 (9.0%) patients.

## Discussion

We report the first *N. gonorrhoeae* AMR surveillance data, quality-assured according to WHO standards [5, 49, 51, 52], for isolates cultured in Belarus during an extended period (from 2009 to 2019 in Minsk, Vitebsk, and Mogilev). AMR levels were significant and comparable to those in neighbouring Russia [41–44], EU/EEA countries [12, 36, 37], and many other countries internationally [5, 52]. However, the AMR levels were clearly higher than in neighbouring Ukraine [33].

In Belarus, the resistance levels to the earlier recommended gonorrhoea therapeutic antimicrobials tetracycline, ciprofloxacin, and benzylpenicillin were relatively high, i.e., at 27.8, 24.7, and 7%, respectively. These antimicrobials should not be recommended or used for empirical gonorrhoea treatment in Belarus, which is in concordance with most other countries [3, 5, 8–12, 28–34, 36, 37, 39–44]. It is a grave concern that fluoroquinolones (ofloxacin and lomefloxacin) remain to be recommended in the 2009 Belarusian national gonorrhoea guideline [45], i.e., despite the high resistance to ciprofloxacin. Furthermore, particularly ofloxacin was also frequently used in gonorrhoea monotherapy or in combination with other antimicrobials. When susceptibility has not been confirmed by laboratory testing, fluoroquinolones should not be used for treatment [8]. Accordingly, ofloxacin and lomefloxacin should be excluded from the empiric treatment recommended in the Belarusian national gonorrhoea guideline [45]. Of note, gonococcal strains producing β-lactamase were very rare (0.2%, 1/522 isolates) in Belarus, as earlier observed also in the neighbouring East European countries Russia and Ukraine [33, 39–44]. Accordingly, few β-lactamase producing strains appear to have emerged in Belarus or, if imported, managed to spread in the country. However, 7% of the 522 isolates had a chromosomally-mediated resistance to benzylpenicillin, and additionally, 36.7% displayed a decreased susceptibility. Azithromycin resistance (0.8%) was rare compared to most EU/EEA countries [12, 36, 37] and only sporadic isolates (*n* = 4) resistant to azithromycin were found. The overall resistance to cefixime was 2.7%, fluctuating from 0 to 22.2% in 2017. Only 36 gonococcal isolates, however, were examined in 2017 and the high cefixime resistance this year was suspected to mainly be caused by a single gonococcal clone. No gonococcal resistance to ceftriaxone, spectinomycin, and gentamicin was identified. However, spectinomycin is currently not available throughout Belarus [46]. The lack of ceftriaxone resistance and, in general, relatively low ceftriaxone MICs may be because of the long tradition to use ceftriaxone in high dose (1 g in monotherapy or dual antimicrobial therapy) for treatment of gonorrhoea [45] and that the less potent oral ESC cefixime has been rarely used for treatment of gonorrhoea in Belarus. Alarmingly, the compliance with the 2009 Belarusian national gonorrhoea guideline [45] was relatively low and many non-compliant suboptimal antimicrobials were prescribed to a large proportion of patients. This lack of compliance may select AMR in *N. gonorrhoeae*, etiological agents of other STIs, and bystander organisms [3, 53]. Moreover, in Belarus antimicrobials, such as several penicillins and tetracyclines, are readily available over-the-counter in pharmacies without prescription, which is crucial to abandon to decrease the high level of self-medication and a further selection of AMR.

Our study has some limitations. First, gonococcal isolates were collected in only three of the six regions of Belarus. However, considering that these three regions represent 60% of the Belarusian population, including the capital city Minsk (https://en.wikipedia.org/wiki/Regions_of_Belarus), this geographical bias should be limited and the results should be generalisable to the wider population. Second, the number of isolates per year was low. Third, no data were collected on pharyngeal or rectal specimens. Finally, no data on epidemiological or clinical characteristics (e.g., sexual behaviour and treatment outcomes) were available. Consequently, the gonococcal AMR surveillance in Belarus should be further improved and enhanced by increasing the number of isolates and representativeness of isolates collected each year, ideally including additional regions and collection of extragenital specimens such as pharyngeal and rectal specimens. Finally, gonococcal AMR surveillance in Belarus should also include more detailed patient epidemiological and clinical data.

## Conclusions

The present study reports the first gonococcal AMR surveillance data for isolates cultured in Belarus over an extended period (i.e., from 2009 to 2019) in the Minsk, Mogilev, and Vitebsk regions, quality-assured according to WHO standards [5, 49, 51, 52]. Based on the gonococcal AMR data presented in this paper, Belarus has also participated in the WHO Global GASP [5]. Briefly, in 2009–2019 the gonococcal population circulating in Belarus showed stable and high resistance to tetracycline, ciprofloxacin, and benzylpenicillin. More sporadic resistance to azithromycin and fluctuating resistance to cefixime were also found. However, no resistance to ceftriaxone, spectinomycin, or gentamicin was identified. Consequently, ceftriaxone 1 g can continuously be recommended as empiric first-line gonorrhoea therapy in Belarus. When susceptibility has not been confirmed by laboratory testing, fluoroquinolones should not be used for gonorrhoea treatment. Continued, improved and enhanced *N. gonorrhoeae* AMR surveillance of gonococcal AMR in Belarus is imperative to timely inform revisions of the national gonorrhoea guideline [45] in Belarus. Finally, a comparison of the Belarusian gonococcal population with the *N. gonorrhoeae* strains spreading internationally, i.e., using whole-genome sequencing, which is used in the Euro-GASP surveillance [37, 54] and has also been recently applied in gonococcal AMR surveillance in Ukraine [55] and many other countries across the world [56–62], would be valuable.

## Data Availability

The datasets used or analysed during the current study are available from the corresponding author on reasonable request.

## Abbreviations

AMR: Antimicrobial resistance
ECOFF: Epidemiological cut-off value
ESC: Extended-spectrum cephalosporin
EU/EEA: European Union/European Economic Area
EUCAST: European Committee on Antimicrobial Susceptibility Testing
Euro-GASP: European Gonococcal Antimicrobial Surveillance Programme
IM: Intramuscularly
IV: Intravenously
MIC: Minimum inhibitory concentration
WHO: World Health Organization
2009 Belarusian national gonorrhoea guideline: Clinical protocol for the diagnosis and treatment of patients with sexually transmitted infections approved by order of the Ministry of Health of the Republic of Belarus 10/29/2009, No. 1020.
